# Tai Chi for fall prevention and balance improvement in older adults: a systematic review and meta-analysis of randomized controlled trials

**DOI:** 10.3389/fpubh.2023.1236050

**Published:** 2023-09-01

**Authors:** Weidong Chen, Min Li, Hai Li, Yanzhao Lin, Zhoushan Feng

**Affiliations:** ^1^Department of Integrated Traditional Chinese and Western Medicine Nutrition, The Sixth Affiliated Hospital, School of Medicine, South China University of Technology, Foshan, China; ^2^The Second Clinical Medical College of Guangzhou University of Chinese Medicine, Guangzhou, China; ^3^College and Hospital of Stomatology, Guangxi Medical University, Nanning, China; ^4^Guangdong Provincial Key Laboratory of Major Obstetric Diseases, Guangdong-Hong Kong-Macao Greater Bay Area Higher Education Joint Laboratory of Maternal-Fetal Medicine, Department of Obstetrics and Gynecology, Guangdong Provincial Clinical Research Center for Obstetrics and Gynecology, Guangzhou, China; ^5^Guangzhou Key Laboratory of Neonatal Intestinal Diseases, Department of Neonatology, The Third Affiliated Hospital of Guangzhou Medical University, Guangzhou, China

**Keywords:** Tai Chi, fall, balance, older adults, meta-analysis

## Abstract

**Background and objective:**

As the population ages, the health of older adults is becoming a public health concern. Falls are a significant threat to their health due to weakened balance. This study aims to investigate the beneficial effects of Tai Chi on fall prevention and balance improvement in older adults.

**Methods:**

We conducted a systematic review and meta-analysis of randomized controlled trials related to Tai Chi, falls, and balance ability, searching PubMed, Embase, and Cochrane Library databases from their establishment until December 31, 2022. Two independent reviewers performed the search, screening of results, extraction of relevant data, and assessment of study quality. This study followed the PRISMA guidelines for systematic review and meta-analysis.

**Results:**

Totally 24 RCTs were included for meta-analysis, and the results showed that Tai Chi can effectively reduce the risk of falls in older adults (RR: 0.76, 95% CI: 0.71 to 0.82) and decrease the number of falls (MD [95% CI]: −0.26 [−0.39, −0.13]). Tai Chi can also improve the balance ability of older adults, such as the timed up and go test (MD [95% CI]: −0.69 [−1.09, −0.29]) and the functional reach test (MD [95% CI]: 2.69 [1.14, 4.24]), as well as other balance tests such as single-leg balance test, Berg balance scale, and gait speed (*p* < 0.05). Subgroup analysis showed that Tai Chi is effective for both healthy older adults and those at high risk of falls (*p* < 0.001), and its effectiveness increases with the duration and frequency of exercise. In addition, the effect of Yang-style Tai Chi is better than that of Sun-style Tai Chi.

**Conclusion:**

Tai Chi is an effective exercise for preventing falls and improving balance ability in older adults, whether they are healthy or at high risk of falling. The effectiveness of Tai Chi increases with exercise time and frequency. Yang-style Tai Chi is more effective than Sun-style Tai Chi.

**Systematic review registration:**

https://clinicaltrials.gov/, identifier CRD42022354594.

## 1. Introduction

The global population is currently experiencing an aging trend, and it is predicted that by the mid-21st century, individuals aged 60 years and above will constitute approximately 20% of the total population (1, 2). As individuals age, physiological changes are inevitable, leading to challenges such as decreased balance ability, weakened muscle strength, and a higher risk of falling (3). Falls are a primary cause of injuries among the older adult, which can result in severe consequences such as fractures, head injuries, and even death, placing a significant burden on the public health system (4). Annually, between 28 and 35% of individuals aged 65 years and older experience falls worldwide, with rates reaching 32–42% among those over 70 years of age (5). Therefore, preventing falls has become a critical global objective for the older adult population.

Tai Chi is a distinctive form of exercise that involves movements primarily performed in a semi-squatting position. These movements require a continuous shift in the body’s center of gravity, incorporating posture control, trunk rotation, weight transfer, and strength training. All these features are advantageous for improving balance and strength, reducing the risk of falling and the fear of falling (6–8). In comparison to other types of exercise, Tai Chi practice has fewer requirements for equipment, venues, and caregivers, making it a more accessible form of exercise to promote (9).

However, previous recommendations regarding Tai Chi as an effective tool for reducing the risk of falling may be limited due to a small number of selected studies, a lack of subgroup analysis on factors such as Tai Chi style, exercise time and frequency, and balance ability analysis (10, 11). Considering the insufficient information available on the impact of Tai Chi on fall prevention among the older adult, this current systematic review aims to explore the recent randomized controlled trials (RCTs) that analyze the effective reduction of fallers, fall rates, and improvement of balance ability in the older adult through the practice of Tai Chi. This systematic review provides valuable insights for using Tai Chi as an intervention for fall prevention in the older adult.

## 2. Methods

Our systematic review was performed following the guidelines of the Preferred Reporting Items for Systematic Reviews and Meta-Analyses (PRISMA) (12) and was registered with PROSPERO using the registration number CRD42022354594.

### 2.1. Search strategy

We conducted a search of databases such as PubMed, EMBASE, and the Cochrane Library using a specific search strategy for each database, covering literature from inception up to December 31, 2022. Additionally, we conducted a review of the references of each included study to ensure that no relevant papers were missed during the search. The full search strategy is available in Supplementary Table S1.

### 2.2. Inclusion criteria for the study

(1) Participants: older adult people aged ≥60 years, as defined by the World Health Organization (13, 14).

(2) Inventions: any form of Tai Chi exercise.

(3) Controls: receiving either exercise that is stretching or other low-level exercises, usual care, or wellness education.

(4) Outcomes: the number of fallers (participants who experienced at least one fall), average number of falls per person, and balance assessment indicators including the timed up and go (TUG) test, functional reach test (FRT), single leg balance test (SLB), Berg balance scale (BBS), short physical performance battery (SPPB) score, fall efficacy scale (FES), and gait speed assessment. Among these, FRT is a simple, practical, and widely used clinical tool to assess an individual’s balance capability and risk of falling. In this test, the maximum distance an individual can reach forward while maintaining a fixed support base in a standing position is measured.

(5) Study: only include RCTs in English.

### 2.3. Management of the study and retrieval of data

After eliminating duplicate studies using Endnote software, two reviewers (W.C. and M.L.) independently selected eligible studies and extracted relevant data. In case of any discrepancies, the reviewers reached a consensus through discussion and negotiation. The collected data included the author’s name, year of publication, sample size, older adult population, health status, age, Tai Chi style, frequency, total exercise time, and follow-up time and outcomes.

### 2.4. Assessment of study quality

The assessment parameters for the evaluation of study quality included random sequence generation, allocation concealment, blinding of participants and assessors, handling of incomplete outcome data, selective reporting, and other biases. Each item was assessed as having “low risk,” “unclear risk,” or “high risk.” Two reviewers (W.C. and M.L.) independently assessed the data using the Cochrane Risk of Bias tool (15) and resolved any discrepancies through mutual discussion based on justifications.

### 2.5. Data analysis

The effect size of binary variables was represented by the risk ratio (RR) with a 95% confidence interval (CI), and the effect size of continuous variables was represented by the mean difference (MD) with a 95% CI, evaluated using a random-effects model. The I^2^ statistic was used to evaluate the heterogeneity among studies, where I^2^ < 50% indicated low heterogeneity and I^2^ ≥ 50% indicated significant heterogeneity. Subgroup analysis was performed on exposure time, Tai Chi style, weekly frequency, risk of fall, and follow-up time.

Sensitivity analysis was performed by using the one-by-one exclusion method and excluding studies with a small number of participants to test the stability of the outcomes. For each result that included 10 or more original trials, we used funnel plots to test for publication bias, and assessed the symmetry of the funnel plot using Egger’s test and Begg’s test (16, 17). If the funnel plot was asymmetric, we used the trim-and-fill method for adjustment (18). A two-tailed *p* < 0.05 was considered statistically significant. All analyses were conducted using RevMan 5.3 and Stata 14.0 software.

## 3. Results

### 3.1. Study selection and characteristics

**Figure 1:**
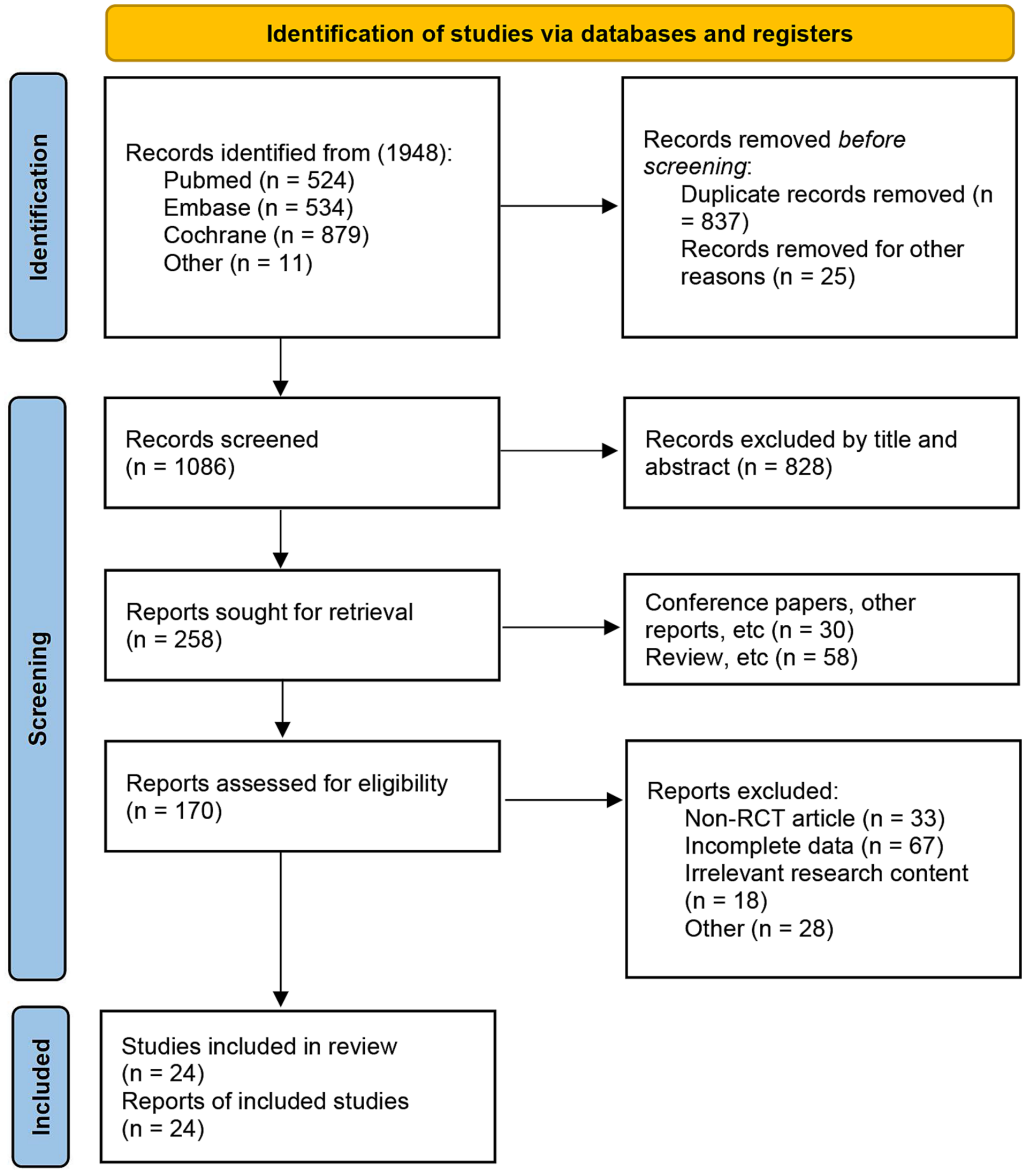
PRISMA study selection flowchart.

A total of 1948 articles were initially searched, of which 837 duplicates and 25 articles without titles were excluded automatically. An additional 828 articles were excluded by reading titles and abstracts. From the remaining 258 articles, 88 were conference papers, other reports, and reviews. Further exclusions were made for 33 non-RCT, 85 articles with incomplete data or unrelated research content, and 28 articles for other reasons. In the end, 24 articles (with one or more outcomes) were included for analysis (7, 8, 19–40) (Figure 1).

Among the 24 articles, 6 reported participants with a history of falls (7, 24, 27, 29, 30, 36), and 5 reported patients with a history of neurological diseases such as stroke or Parkinson’s disease (8, 21, 35, 37, 38). The Tai Chi styles used in the interventions were mainly Sun-style (19, 25, 33, 34, 36, 39) or Yang-style (7, 21, 23, 24, 26, 32, 35, 37, 40), with one article using Chen-style (30) and others not specifying. Exercise frequency ranged from one to three times a week, with one article reporting five times a week (23). Total exercise time was mainly within 72 h, with one article reporting 96 h (19) and another reporting 144 h (40). Follow-up periods were ≤ 3 months in 6 articles (25, 30, 32–35), 3–6 months in 9 articles (8, 20, 23, 26–28, 36, 37, 39), 6–12 months in 7 articles (7, 19, 21, 22, 29, 38, 40), and > 12 months in 2 articles (24, 31) (Table 1).

**Table 1:** Characteristics of included studies.

Study year	Country	Risk of falls	Age	Tai Chi/Control (n)	Intensity	Exposure Time (h)	Follow-up time
Day et al. (19)	Australia	Non-high risk	77.6	Sun style Tai Chi (205)	Twice a week for 48 weeks, 60 min per session.	96	12 months
			77.8	Stretching (204)			
Faber et al. (20)	Netherlands	Non-high risk	84.4	Tai Chi (80)	Once a week for 4 weeks, followed by twice weekly for 16 weeks. 90 min per session	54	5 months
			85.4	Functional walking (66)			
			84.9	No intervention (92)			
Gao et al. (21)	American	Parkinson’s disease and history of falls^#^	69.54	Yang style Tai Chi (40)	Three sessions per week for 12 weeks, 60 min per session.	36	12 months
			68.28	Usual care (40)			
Huang et al. (22)	China	Non-high risk	71.40	Tai Chi (31)	Three sessions per week for 20 weeks,60 min per session.	60	12 months
			71.50	No Tai Chi (47)			
Huang et al. (23)	China	Non-high risk	>60	Yang style Tai Chi (62)	Five sessions per week for 8 weeks, 60 min per session.	40	6 months
				Cognitive Intervention (62)			
				No intervention (62)			
Hwang et al. (24)	China	History of falls	72.0	Yang style Tai Chi (182)	Once a week for 6 months, 60 min per session.	24	18 months
			72.7	Balance training (175)			
Kim et al. (25)	South Korea	Non-high risk	71.4	Sun style Tai Chi (23)	Twice a week for 12 weeks, 60 min per session.	24	3 months
			70.9	Taekwondo (23)			
Li et al. (26)	American	Non-high risk	77.4	Yang-style Tai Chi (125)	Three times per week for 6 months, 60 min per session.	72	6 months
			77.4	Stretching (131)			
Li et al. (8)	American	Parkinson’s disease^￥^	68	Tai Chi (65)	Twice a week for 24 weeks, 60 min per session.	48	6 months
			69	Resistance (65)			
			69	Stretching (65)			
Li et al. (27)	American	History of falls	77.5	Tai Chi (224)	Twice a week for 24 weeks, 60 min per session.	48	6 months
			77.8	Multimodal Exercise (223)			
			77.8	Stretching (223)			
Li et al. (28)	American	Non-high risk	76.13	Tai Chi (15)	Three sessions per week for 24 weeks, 60 min per session.	72	6 months
			76.20	Stretching (15)			
Logghe et al. (29)	Netherlands	History of falls	77.5	Yang-style Tai Chi (138)	Twice a week for 13 weeks, 60 min per session.	26	12 months
			76.8	Usual care (131)			
Ni et al. (30)	American	History of falls	70.27	Chen-style Tai Chi (11)	Twice a week for 12 weeks, 60 min per session.	24	3 months
			77.80	Balance training (15)			
Nowalk et al. (31)	American	Non-high risk	82.8	Tai Chi (38)	Three times per week	–	24 months
			85.5	FNBF exercise (37)			
			85.9	Basic enhanced program (35)			
Penn et al. (32)	China	Non-high risk	75.3	Yang-style Tai Chi (15)	Three sessions per week for 8 weeks, 30 min per session.	12	2 months
			73.4	Wellness education (15)			
Pluchino et al. (33)	American	Non-high risk	69.3	Sun-style Tai Chi (14)	Twice a week for 8 weeks, 60 min per session.	16	2 months
			76.0	Standard balance item (14)			
Son et al. (34)	South Korea	Non-high risk	72.8	Sun-style Tai Chi (21)	Twice a week for 12 weeks, 60 min per session.	24	3 months
			71.5	Balance training (24)			
Taylor et al. (36)	American	Post-stroke^￥^	72.8	Yang-style Tai Chi (12)	Three sessions per week for 12 weeks, 50 min per session.	30	3 months
			64.5	Usual care (16)			
Taylor et al. (35)	New Zealand	History of falls	75.3	Sun-style Tai Chi (223)	Once/twice a week for 20 weeks, 60 min per session.	20	5 months
			74.4	Sun-style Tai Chi (220)		40	
			73.7	Low-level exercise (231)			
Taylor et al. (37)	American	Post-stroke	71.5	Yang-style Tai Chi (30)	Three sessions per week for 12 weeks, 60 min per session.	36	5 months
			69.6	Usual community-based exercise (31)			
			68.2	Usual care (28)			
Tousignant et al. (38)	Canada	Frail older adults* and history of falls	79.1	Tai Chi (76)	Twice a week for 15 weeks, 60 min per session.	30	12 months
			80.7	Conventional physical therapy (76)			
Voukelatos et al. (39)	Australia	Non-high risk	Mean 69 year	Sun (83%) or Yang-style Tai Chi (353)	Once a week for 16 weeks, 60 min per session	16	4 months
				No Tai Chi (349)			
Wolf et al. (7)	American	History of falls	80.9	Yang-style Tai Chi (145)	Twice a week for 48 weeks, 30 ~ 45 min per session	48–64	12 months
			80.9	Wellness education (141)			
Woo et al. (40)	China	Non-high risk	68.9	Yang-style Tai Chi (60)	Three times per week for 12 months，60 min per session.	144	12 months
			68.8	Resistance training (60)			
			68.1	No intervention (60)			

*Types of diagnoses varied from stroke/neurological diseases, musculoskeletal and gait disorders.

^#^At least one fall in the past 12 months.

^￥^Occurred in the past 6 months.

### 3.2. Evaluating the risk of bias

**Figure 2:**
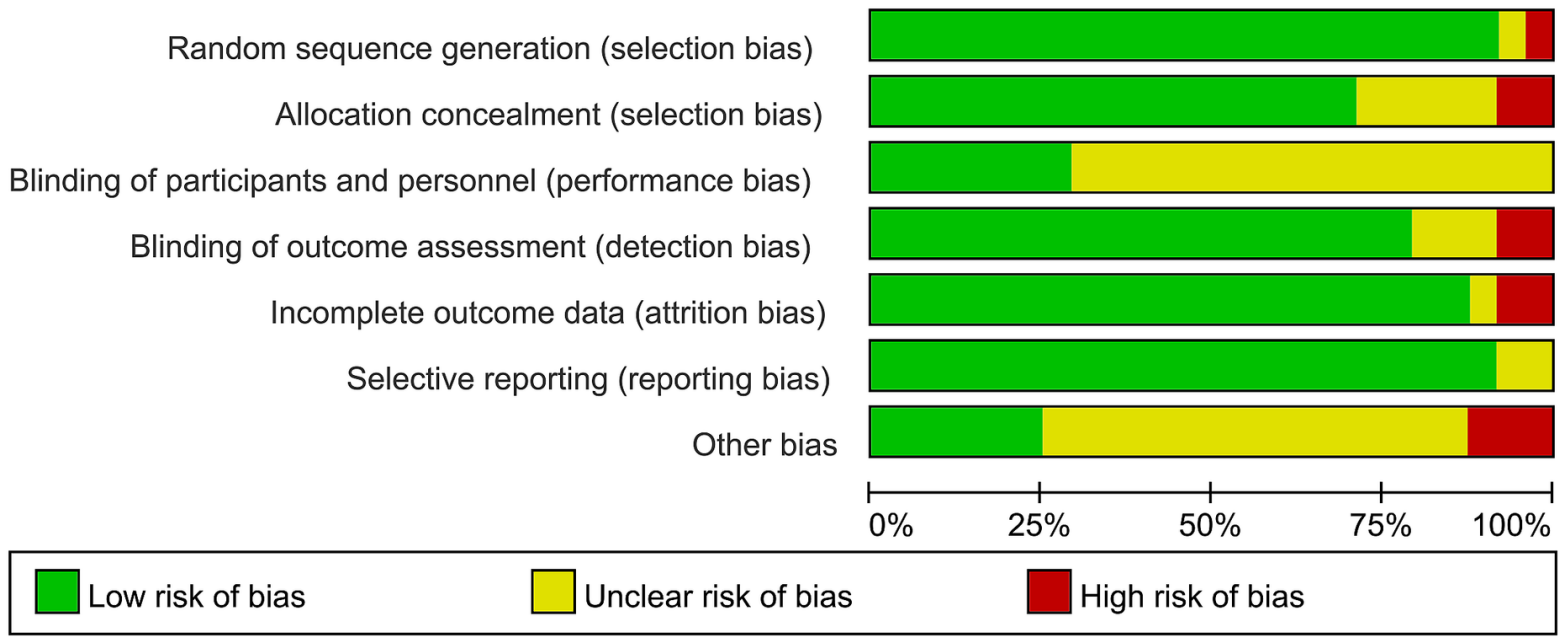
Risk of bias graph.

Due to the appropriate methods used in random sequence generation and allocation concealment, 22 (8, 19–31) or 17 (8, 19, 20, 23–28, 31, 33, 35–40) articles were considered low risk, respectively. In addition, only 7 articles (7, 8, 20, 26, 29, 36, 38) were considered low risk due to the difficulty in blinding participants, while 19 articles used blinding well in assessing outcomes (7, 8, 19–21, 23–29, 32, 34–37, 39, 40). In terms of incomplete outcome data and selective reporting, 21 (8, 19–21, 23–35, 37–40) and 22 (8, 19–27, 29–32) articles were considered low bias, respectively (Figure 2 and Supplementary Figure S1).

### 3.3. Number of fallers

**Figure 3:**
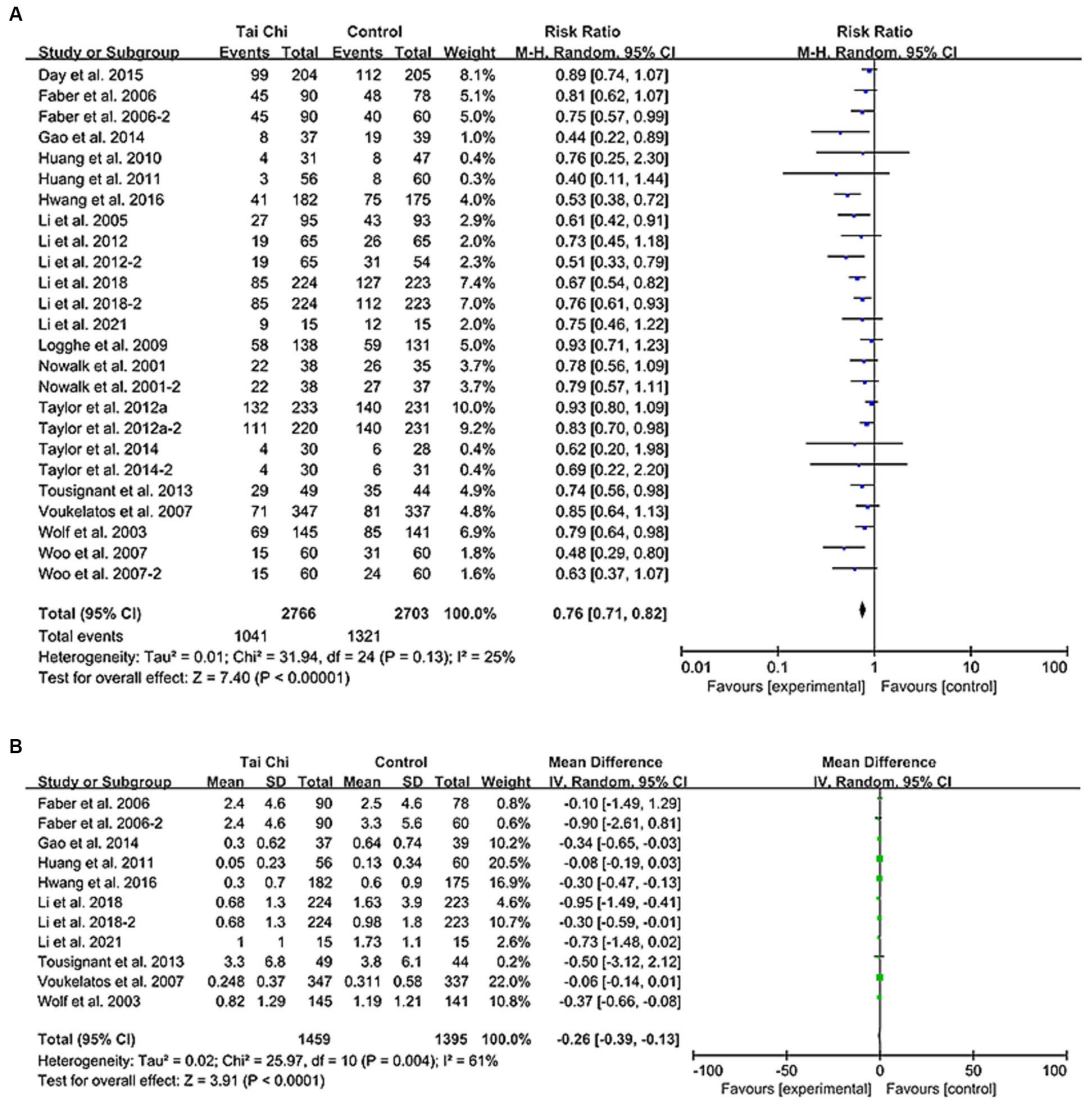
Forest plot comparing Tai Chi with control group. **(A)** Number of fallers. **(B)** Rate of falls. CI, confidence interval; RR, risk ratio.

A total of 18 studies provided data on the number of fallers (7, 8, 19–24, 26–29, 31, 36–40), and meta-analysis found that Tai Chi exercise could significantly reduce the number of fallers (1,041/2766 VS. 1,321/2703; RR: 0.76, 95% CI: 0.71–0.82, I^2^: 25%, *p* < 0.001) (Figure 3A). Sensitivity analysis was performed by sequentially excluding each trial or removing studies with fewer than 100 participants, and the results showed that there was no significant change in statistical significance and heterogeneity. Subgroup analysis showed that Tai Chi could prevent falls in any population at risk, regardless of the overall duration of Tai Chi exercise, follow-up time, or Tai Chi style (Sun or Yang) (all *p* < 0.01). However, in terms of exercise frequency, exercising twice (7, 8, 19, 20, 27, 29, 36, 38) or ≥ 3 times (21–23, 26, 28, 31, 37, 40) per week showed more significant benefits than exercising once a week (RR [95% CI] were 0.78 [0.73, 0.84] and 0.67 [0.58, 0.79], respectively) (Table 2).

**Table 2:** Subgroup analysis of fallers.

Subgroup	Included studies	Tai Chi group positive/total	Control group positive/total	Heterogeneity (I^2^)	RR [95% CI]	*p*	Test for subgroup difference
Exposure time (h)							0.69
≤24	4 (23, 24, 37, 39)	247/818	304/803	75%	0.74 [0.53, 1.02]	0.06	
24 ~ 48	7 (8, 21, 27, 29, 36–38)	422/1082	561/1069	15%	0.74 [0.68, 0.82]	<0.001	
>48	7 (7, 19, 20, 22, 26, 28, 40)	328/790	403/759	0	0.76 [0.69, 0.85]	<0.001	
Tai Chi style							0.004
Sun	3 (19, 37, 39)	413/1004	473/1004	0%	0.88 [0.81, 0.97]	0.008	
Yang	9 (7, 21, 23, 24, 26, 29, 37, 40)	244/833	829/1822	36%	0.76 [0.68, 0.86]	<0.001	
Weekly frequency							0.22
Once	3 (24, 36, 39)	244/762	296/743	81%	0.76 [0.55, 1.06]	0.11	
Twice	8 (7, 8, 19, 20, 27, 29, 36, 38)	664/1514	815/1455	4%	0.78 [0.73, 0.84]	<0.001	
≥Three times	8 (21–23, 26, 28, 31, 37, 40)	133/490	210/505	0	0.67 [0.58, 0.79]	<0.001	
Risk of fall							0.18
Non-high risk	9 (19, 20, 22, 23, 26, 28, 31, 39, 40)	377/1124	460/1087	0	0.78 [0.71, 0.86]	<0.001	
History of falls only	5 (7, 24, 27, 29, 35)	581/1366	738/1355	62%	0.78 [0.69, 0.89]	<0.001	
Falls related diseases	4 (8, 21, 37, 38)	83/276	123/261	0	0.42 [0.54, 0.80]	<0.001	
Follow-up time							0.71
≤6 months	9 (8, 20, 23, 26–28, 36, 37, 39)	659/1784	820/1729	15%	0.77 [0.71, 0.84]	<0.001	
6 ~ 12 months	7 (7, 19, 21, 22, 29, 38, 40)	297/724	373/727	32%	0.77 [0.67, 0.89]	<0.001	
>12 months	2 (24, 31)	85/258	1228/247	56%	0.69 [0.51, 0.91]	0.009	

RR, risk ratio.

### 3.4. Rate of falls

According to 15 articles providing the total number of falls, the Tai Chi group had a lower incidence of falls compared to the control group (1816/2539 VS. 2,681/2475, IRR, 0.66) (see Supplementary Table S2) (7, 8, 19–21, 23, 24, 26–29, 36–39). Among them, 9 articles provided mean and standard deviation information (7, 20, 21, 23, 24, 27, 28, 38, 39). Tai Chi significantly reduced the number of falls per person (MD [95%CI]: −0.26 [−0.39, −0.13], I^2^ = 61%, *p* < 0.001) (Figure 3B). Excluding the Li et al. study effectively reduced heterogeneity (I^2^ = 49%), but did not significantly affect the results (−0.12 [−0.17, −0.06], *p* < 0.001) (7, 20, 21, 23, 24, 28, 38, 39). Moreover, studies excluding fewer participants did not significantly change the results (−0.12 [−0.18, −0.07], *p* < 0.001) (7, 20, 23, 24, 38, 39). Subgroup analysis showed that Tai Chi is more effective in reducing falls per person with longer exercise time (*p* = 0.15, 0.02, and < 0.01, respectively), and that Yang-style Tai Chi is more effective than Sun-style Tai Chi (*p* < 0.01 and = 0.09, respectively). Furthermore, regardless of the exercise follow-up duration (≤6 months, 6 ~ 12 months, or > 12 months), Tai Chi exercise was found to significantly reduce the number of falls per person (*p* = 0.01, <0.01, and < 0.01, respectively) (Supplementary Table S3). These findings can be used to design better Tai Chi interventions for fall prevention.

### 3.5. Timed up and go test

**Figure 4:**
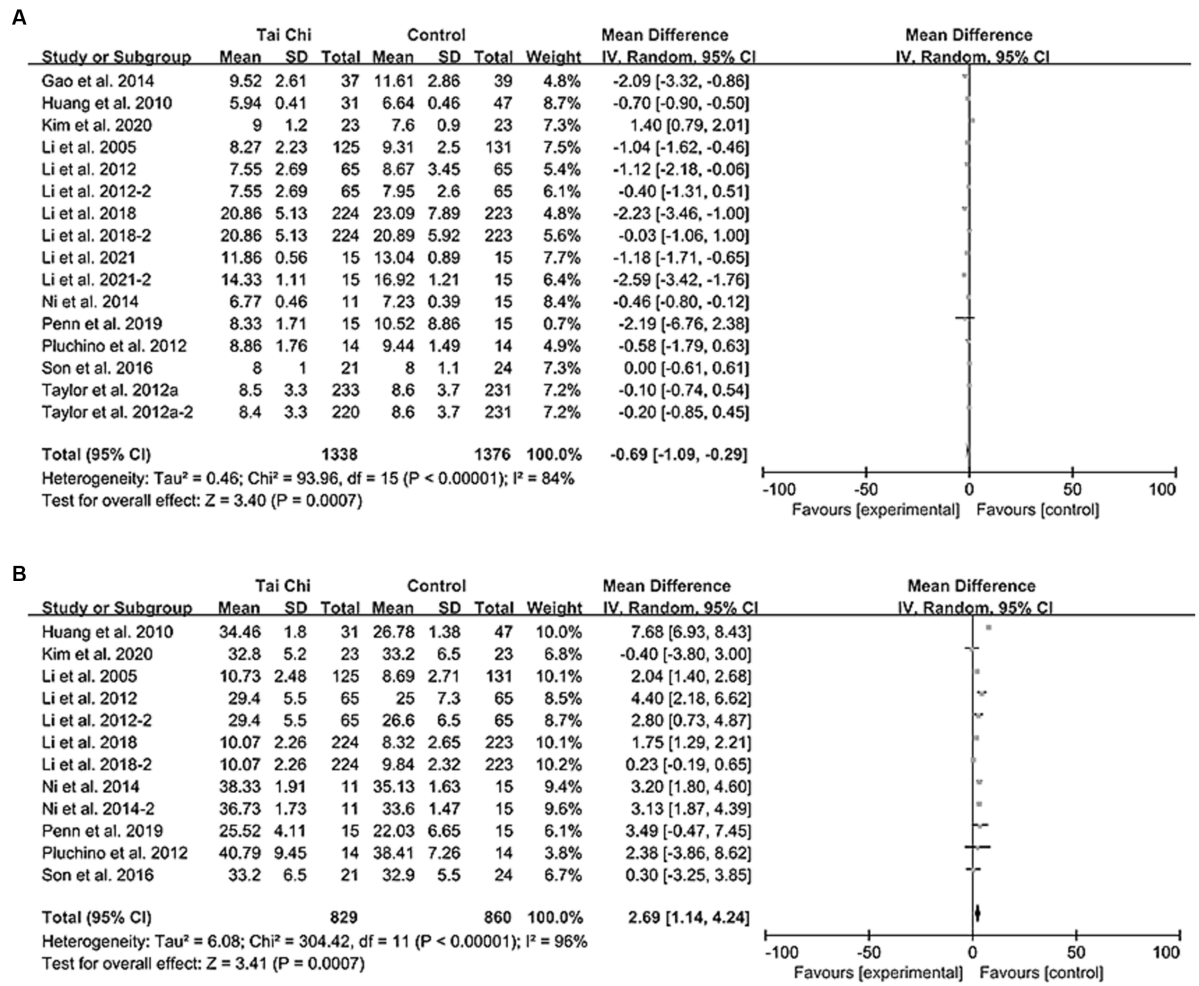
Forest plot comparison of balance ability between Tai Chi group and control group. **(A)** TUG. **(B)** FRT. CI, confidence interval; MD, mean difference; TUG, timed up and go; FRT, functional reach test.

Meta-analysis of 12 studies showed that Tai Chi can significantly reduce TUG time (MD [95%CI]: −0.69 [−1.09, −0.29], *p* < 0.001) (Figure 4A) (8, 21, 22, 25–28, 30, 32–34, 36). Sensitivity analysis was conducted by sequentially deleting each trial and excluding studies with smaller sample sizes, and the significance and heterogeneity of the results remained unchanged. With increasing exercise time and frequency, Tai Chi was more effective in reducing TUG time (*p* = 0.93, 0.01, and <0.001; *p* = 0.76, 0.28, and <0.001, respectively). Yang-style Tai Chi was more effective than Sun-style Tai Chi (*p* < 0.01 and = 0.66, respectively). In addition, Tai Chi can shorten TUG time in older adult people with falls related diseases (*p* = 0.02) (Supplementary Table S4) (21, 38).

### 3.6. Functional reach test

Meta-analysis of 9 studies providing FRT data has shown that the functional reach distance of practicing Tai Chi was significantly greater than that of the control group (MD [95%CI]: 2.69 [1.14, 4.24], *p* < 0.001) (Figure 4B) (8, 22, 25–27, 30, 32–34). Sensitivity analysis, conducted by sequentially excluding each study or studies with smaller sample sizes, did not significantly alter the results (all *p* < 0.01). Subgroup analysis revealed that Yang-style Tai Chi was more effective than Sun-style Tai Chi (*p* < 0.001 and 0.82, respectively), and practicing Tai Chi twice a week had a better effect (*p* < 0.001). Furthermore, Tai Chi was found to be particularly effective in improving FRT test scores in older adult individuals with a history of falls or fall-related diseases (*p* = 0.003 and < 0.001, respectively) (Supplementary Table S5).

### 3.7. Other outcomes

Through a meta-analysis of other fall or balance ability tests, it was found that Tai Chi can effectively lengthen SLB test times (including with eyes open and closed) (MD [95% CI]: 9.63 [5.87, 13.40], *p* < 0.001) (25, 26, 30, 33, 34), increase BBS scores (MD [95% CI]: 1.80 [0.09, 3.51], *p* = 0.04) (21, 26, 29, 32), and improve gait speed (MD [95% CI]: 9.26 [1.00, 17.52], *p* = 0.03) (8, 25, 26, 34). In addition, Tai Chi did not have a significant effect on overall SPPB scores (27, 35, 37) or FES scores (24, 29, 34) (MD [95% CI]: −0.07 [−1.07, 0.93], 0.17 [−0.49, 0.83]; *p* = 0.89, 0.61; respectively) (Table 3).

**Table 3:** Other outcomes.

Outcomes	Included studies	Tai Chi group	Control group	Heterogeneity (I^2^)	MD [95% CI]	*p*
SLB	5 (25, 26, 30, 33, 34)	330	353	69%	9.63 [5.87, 13.40]	<0.001
BBS	4 (21, 26, 29, 32)	315	316	65%	1.80 [0.09, 3.51]	0.04
Gait speed	4 (8, 25, 26, 34)	299	308	91%	9.26 [1.00, 17.52]	0.03
SPPB scores	3 (27, 35, 37)	524	517	90%	−0.07 [−1.07, 0.93]	0.89
FES	3 (24, 29, 34)	334	320	10%	0.17 [−0.49, 0.83]	0.61

MD, mean difference; CI, confidence interval; SLB, single leg balance test; BBS, Berg balance scale; SPPB, short physical performance battery; FES, fall efficacy scale.

### 3.8. Publication bias

**Figure 5:**
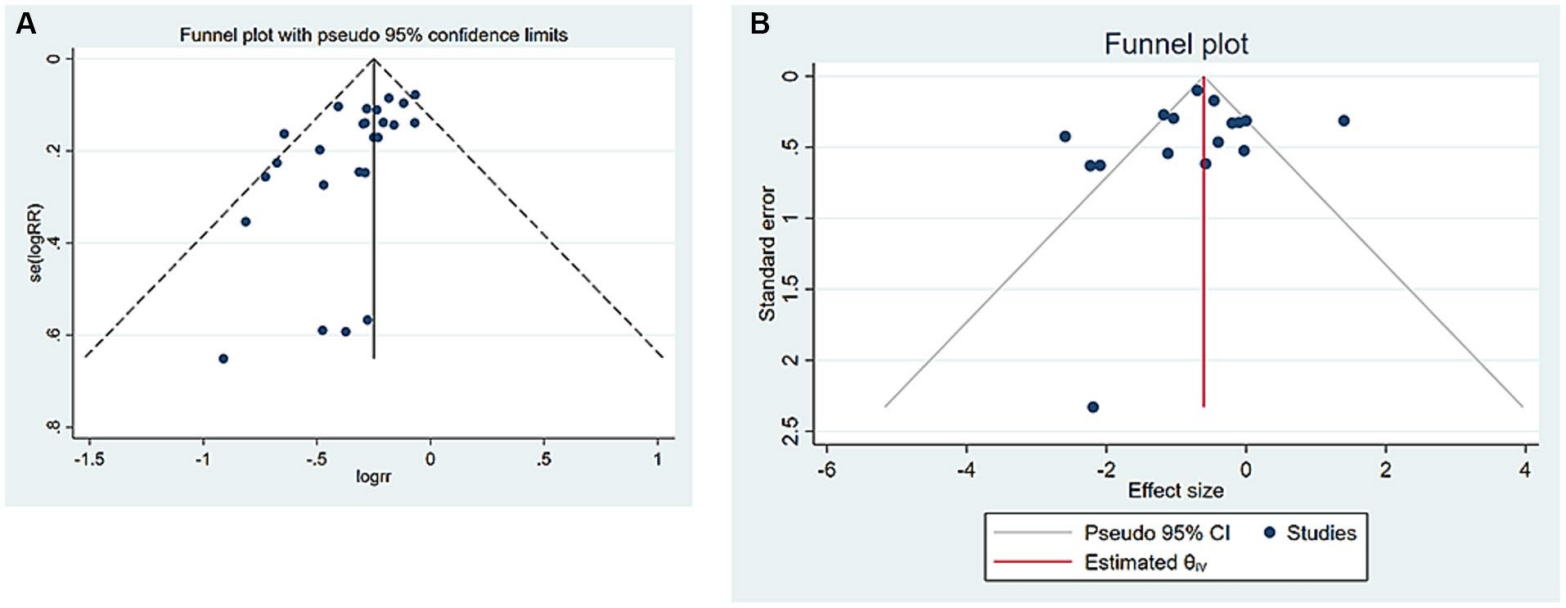
Publication bias. **(A)** Number of fallers. **(B)** TUG. CI, confidence interval; RR, risk ratio; TUG, timed up and go.

Publication bias analyses were conducted on the results of more than 10 studies on fallers and TUG. It was found that there was a significant publication bias in the Fallers results (Begg’s test: 0.014 and Egger’s test: 0.003) (Figure 5A). The trim-and-fill method was used to adjust for publication bias, and the outcome still showed statistical differences (*p* < 0.001). In addition, there was no significant publication bias in the TUG results (Begg’s test: 0.092, Egger’s test: 0.315) (Figure 5B).

## 4. Discussion

In the meta-analysis, Tai Chi exercise can effectively decrease the rate of fallers and number of falls in older adults, including those with a high risk of falling, while also improving their balance, as measured by TUG, FRT, SLB, BBS score, or gait speed. The effectiveness of Tai Chi increases with the duration and frequency of exercise. Furthermore, Yang-style Tai Chi was found to be more effective than Sun-style Tai Chi. Sensitivity analysis by excluding individual studies and those with small sample sizes did not significantly alter the outcomes.

The importance of Tai Chi for older adults is increasingly recognized in research. Past meta-analyses have found that Tai Chi can improve cognitive abilities (41), enhance the quality of life and alleviate symptoms related to cancer (42), and relieve chronic pain (43), among other benefits. Our study results, in comparison to previous systematic reviews, consistently find that Tai Chi is indeed effective in preventing falls among the older adult. However, there is a lack of exploration regarding the results of improving balance and the relationship between improving balance and preventing falls (4, 44, 45). By including more RCTs, our research not only investigates Tai Chi’s role in fall prevention but also explores whether it is related to enhancing balance abilities. Moreover, our study adds more nuances to these findings. For example, the differences in various Tai Chi practices, exercise durations, and frequencies, could guide future Tai Chi exercise instructions more effectively.

Compared to other exercise interventions or non-interventions, Tai Chi is an effective fall prevention method for the older adult and has been validated in previous studies (10, 11, 44). This study included more updated randomized controlled trials and examined the impact of Tai Chi on balance ability. The fall prevention effect of Tai Chi may stem from its ability to improve balance (7, 21, 26). The study found that Tai Chi can not only improve static balance (e.g., SLB), but also dynamic balance (e.g., TUG and gait speed), and enhance postural control (e.g., FRT). Tai Chi exercise conforms to the statics balance theory, where the main factors affecting human balance are the size of the support surface and the height of the center of gravity (46). Therefore, Tai Chi can improve the ability of the older adult to control their center of gravity and adjust their posture, thereby improving balance ability (47, 48).

Tai Chi movements require a semi-squat position and involve concentric and eccentric contractions of leg muscles at varying degrees during practice, which has been reported to significantly enhance lower limb strength and endurance in the older adult (49, 50). Tai Chi practice also provides moderate aerobic exercise and flexibility training, which can improve cognitive function in older adults (41, 51). The enhancement of muscle strength is associated with the improvement of physical fitness and mental quality of life in the older adult (52). Compared to stretching training, Tai Chi’s precise joint control and muscle coordination helps achieve better balance control (53). Additionally, Tai Chi’s unique meditation component can improve attention and cognitive function, enhancing body control and balance ability in older adults (54). Therefore, Tai Chi’s unique exercise characteristics are an important factor in its success in fall prevention among the older adult, reducing hospitalization rates associated with falls in the community (55). These mechanisms may explain Tai Chi’s role in enhancing balance ability and preventing falls, with significant effects observed over certain follow-up periods. Although some studies have suggested that the protective effect of Tai Chi on fall prevention in the short-term appears to be greater than in the long-term, indicating a potential loss of effectiveness over time (11), this may be due to inconsistencies in the duration of Tai Chi exercise and follow-up time in some studies. It was discovered in our study that Tai Chi’s improvement in balance ability and fall prevention effectiveness increases with longer exercise duration and frequency. A meta-analysis showed that the optimal total exercise time is between 50 and 72 h with 3 exercise sessions per week (56). However, the optimal exercise frequency and duration per week remain unclear. This necessitates further in-depth research on our part.

This study found that Tai Chi is effective not only for healthy older adults but also for those at high risk of falling. In studies on stroke and Parkinson’s disease patients, Tai Chi was found to improve balance ability and prevent falls after stroke, and Tai Chi training was effective in improving the motor ability of stroke patients (48, 56). Further research is needed to evaluate Tai Chi as an adjunctive rehabilitation method, an effective alternative rehabilitation option, or a maintenance strategy. Additionally, Tai Chi can improve cognitive function and immunity in older adults (57, 58). Cost–benefit studies of fall prevention strategies have shown that Tai Chi is the most cost-effective exercise among older adults living in the community (59). Therefore, Tai Chi’s role in the older adult population is universal.

The strengths of our study lie in the inclusion of more recent RCT studies for analysis, the implementation of multiple subgroup analyses based on study characteristics, and the strict execution of sensitivity analyses. However, our study is not without limitations. Firstly, our selection of only English-language literature and specific criteria-based studies may have introduced selection bias. Secondly, the results of our research are only applicable to standard Tai Chi classes, not personalized courses, indicating a need for further investigation into the effects of personalized Tai Chi courses on older adult rehabilitation. Thirdly, despite conducting sensitivity and subgroup analyses, potential heterogeneity across studies may still impact the stability of our results. Lastly, our study only compares Tai Chi exercises with traditional non-Tai Chi exercises, yet a myriad of emerging technologies designed to enhance the quality of life for the older adult warrant further comparative research and exploration in the future (60).

Overall, Tai Chi exercise has a good preventive effect on falls in the older adult (both healthy and high-risk for falls) and enhances their balance ability. However, there is a relatively small number of studies on balance ability, and further research is needed in this area. Additionally, we need to increase the frequency of follow-up observations to observe the process of the Tai Chi intervention’s effectiveness. Furthermore, due to limitations in the number and quality of included studies, the above conclusions still require further high-quality verification.

## Data availability statement

The original contributions presented in the study are included in the article/Supplementary material, further inquiries can be directed to the corresponding author/s.

## Author contributions

WC and ML made equal contributions to this study, including designing the search strategy for retrieving and screening articles, conducting data extraction and analysis. WC and HL wrote the manuscript, while YL and ZF managed the project and reviewed the manuscript. All authors have read and approved the final version of the manuscript.

## Conflict of interest

The authors declare that the research was conducted in the absence of any commercial or financial relationships that could be construed as a potential conflict of interest.

## Publisher’s note

All claims expressed in this article are solely those of the authors and do not necessarily represent those of their affiliated organizations, or those of the publisher, the editors and the reviewers. Any product that may be evaluated in this article, or claim that may be made by its manufacturer, is not guaranteed or endorsed by the publisher.

## Supplementary material

Supplementary Figure 1 Risk of bias summary.

## References

[1] Beard JR Officer A de Carvalho IA Sadana R Pot AM Michel JP . The world report on ageing and health: a policy framework for healthy ageing. Lancet. (2016) 387:2145–54. doi: 10.1016/S0140-6736(15)00516-4, PMID: 26520231 PMC4848186

[2] Chen Y Yang C Li N Wang Z Wu P Du J . Effects of population aging on the mortality burden of related cancers in urban and rural areas of China, 2004-2017: a population-based study. Cancer Biol Med. (2022) 19:696–706. doi: 10.20892/j.issn.2095-3941.2021.0538, PMID: 35235277 PMC9196052

[3] Delbaere K Close JC Brodaty H Sachdev P Lord SR. Determinants of disparities between perceived and physiological risk of falling among elderly people: cohort study. BMJ. (2010) 341:c4165. doi: 10.1136/bmj. 20724399 PMC2930273

[4] Gillespie LD Robertson MC Gillespie WJ Sherrington C Gates S Clemson LM . Interventions for preventing falls in older people living in the community. Cochrane Database Syst Rev. (2012) 2021:CD007146. doi: 10.1002/14651858.CD007146.pub3, PMID: 22972103 PMC8095069

[5] Rubenstein LZ. Falls in older people: epidemiology, risk factors and strategies for prevention. Age Ageing. (2006) 35:ii37–41. doi: 10.1093/ageing/afl084 16926202

[6] Li JX Hong Y Chan KM. Tai Chi: physiological characteristics and beneficial effects on health. Br J Sports Med. (2001) 35:148–56. doi: 10.1136/bjsm.35.3.148, PMID: 11375872 PMC1724328

[7] Wolf SL Sattin RW Kutner M O'Grady M Greenspan AI Gregor RJ. Intense Tai Chi exercise training and fall occurrences in older, transitionally frail adults: a randomized, controlled trial. J Am Geriatr Soc. (2003) 51:1693–701. doi: 10.1046/j.1532-5415.2003.51552.x, PMID: 14687346

[8] Li F Harmer P Fitzgerald K Eckstrom E Stock R Galver J . Tai Chi and postural stability in patients with Parkinson's disease. N Engl J Med. (2012) 366:511–9. doi: 10.1056/NEJMoa1107911, PMID: 22316445 PMC3285459

[9] Chen X Savarese G Cai Y Ma L Lundborg CS Jiang W . Tai Chi and Qigong practices for chronic heart failure: a systematic review and meta-analysis of randomized controlled trials. Evid Based Complement Alternat Med. (2020) 2020:1–15. doi: 10.1155/2020/2034625 PMC7755480 33381195

[10] Del-Pino-Casado R Obrero-Gaitan E Lomas-Vega R. The effect of Tai Chi on reducing the risk of falling: a systematic review and meta-analysis. Am J Chin Med. (2016) 44:895–906. doi: 10.1142/S0192415X1650049X, PMID: 27430918

[11] Lomas-Vega R Obrero-Gaitan E Molina-Ortega FJ Del-Pino-Casado R. Tai Chi for risk of falls. A meta-analysis. J Am Geriatr Soc. (2017) 65:2037–43. doi: 10.1111/jgs.15008, PMID: 28736853

[12] Liberati A Altman DG Tetzlaff J Mulrow C Gotzsche PC Ioannidis JP . The PRISMA statement for reporting systematic reviews and meta-analyses of studies that evaluate healthcare interventions: explanation and elaboration. BMJ. (2009) 339:b2700. doi: 10.1136/bmj.b2700, PMID: 19622552 PMC2714672

[13] World Health Organization. Ageing and health. Available at: https://wwwwhoint/news-room/fact-sheets/detail/ageing-and-health (2021).

[14] Yen FS Wang SI Lin SY Chao YH Wei JC. The impact of heavy alcohol consumption on cognitive impairment in young old and middle old persons. J Transl Med. (2022) 20:155. doi: 10.1186/s12967-022-03353-3, PMID: 35382817 PMC8981936

[15] Cumpston M Li T Page MJ Chandler J Welch VA Higgins JP . Updated guidance for trusted systematic reviews: a new edition of the Cochrane handbook for systematic reviews of interventions. Cochrane Database Syst Rev. (2019) 10:ED000142. doi: 10.1002/14651858.ED000142, PMID: 31643080 PMC10284251

[16] Begg CB Mazumdar M. Operating characteristics of a rank correlation test for publication bias. Biometrics. (1994) 50:1088–101. doi: 10.2307/2533446 7786990

[17] Egger M Davey Smith G Schneider M Minder C. Bias in meta-analysis detected by a simple, graphical test. BMJ. (1997) 315:629–34. doi: 10.1136/bmj.315.7109.629, PMID: 9310563 PMC2127453

[18] Duval S Tweedie R. Trim and fill: a simple funnel-plot-based method of testing and adjusting for publication bias in meta-analysis. Biometrics. (2000) 56:455–63. doi: 10.1111/j.0006-341x.2000.00455.x, PMID: 10877304

[19] Day L Hill KD Stathakis VZ Flicker L Segal L Cicuttini F . Impact of Tai-Chi on falls among preclinically disabled older people. A randomized controlled trial. J Am Med Dir Assoc. (2015) 16:420–6. doi: 10.1016/j.jamda.2015.01.089, PMID: 25769960

[20] Faber MJ Bosscher RJ Chin APMJ van Wieringen PC. Effects of exercise programs on falls and mobility in frail and pre-frail older adults: a multicenter randomized controlled trial. Arch Phys Med Rehabil. (2006) 87:885–96. doi: 10.1016/j.apmr.2006.04.005, PMID: 16813773

[21] Gao Q Leung A Yang Y Wei Q Guan M Jia C . Effects of Tai Chi on balance and fall prevention in Parkinson's disease: a randomized controlled trial. Clin Rehabil. (2014) 28:748–53. doi: 10.1177/0269215514521044, PMID: 24519923

[22] Huang HC Liu CY Huang YT Kernohan WG. Community-based interventions to reduce falls among older adults in Taiwan - long time follow-up randomised controlled study. J Clin Nurs. (2010) 19:959–68. doi: 10.1111/j.1365-2702.2009.02834.x, PMID: 20492040

[23] Huang TT Yang LH Liu CY. Reducing the fear of falling among community-dwelling elderly adults through cognitive-behavioural strategies and intense Tai Chi exercise: a randomized controlled trial. J Adv Nurs. (2011) 67:961–71. doi: 10.1111/j.1365-2648.2010.05553.x, PMID: 21214623

[24] Hwang HF Chen SJ Lee-Hsieh J Chien DK Chen CY Lin MR. Effects of home-based Tai Chi and Lower extremity training and self-practice on falls and functional outcomes in older fallers from the emergency department-a randomized controlled trial. J Am Geriatr Soc. (2016) 64:518–25. doi: 10.1111/jgs.13952, PMID: 26865039

[25] Kim CY Je HD Jeong H Jeong JH Kim HD. Effects of Tai Chi versus Taekkyon on balance, lower-extremity strength, and gait ability in community-dwelling older women: a single-blinded randomized clinical trial. J Back Musculoskelet Rehabil. (2020) 33:41–8. doi: 10.3233/BMR-181493, PMID: 31282402

[26] Li F Harmer P Fisher KJ McAuley E Chaumeton N Eckstrom E . Tai Chi and fall reductions in older adults: a randomized controlled trial. J Gerontol A Biol Sci Med Sci. (2005) 60:187–94. doi: 10.1093/gerona/60.2.187, PMID: 15814861

[27] Li F Harmer P Fitzgerald K Eckstrom E Akers L Chou LS . Effectiveness of a therapeutic *Tai Ji Quan* intervention vs a multimodal exercise intervention to prevent falls among older adults at high risk of falling: a randomized clinical trial. JAMA Intern Med. (2018) 178:1301–10. doi: 10.1001/jamainternmed.2018.3915, PMID: 30208396 PMC6233748

[28] Li F Harmer P Voit J Chou LS. Implementing an online virtual falls prevention intervention during a public health pandemic for older adults with mild cognitive impairment: a feasibility trial. Clin Interv Aging. (2021) 16:973–83. doi: 10.2147/CIA.S306431, PMID: 34079243 PMC8164667

[29] Logghe IH Zeeuwe PE Verhagen AP Wijnen-Sponselee RM Willemsen SP Bierma-Zeinstra SM . Lack of effect of Tai Chi Chuan in preventing falls in elderly people living at home: a randomized clinical trial. J Am Geriatr Soc. (2009) 57:70–5. doi: 10.1111/j.1532-5415.2008.02064.x, PMID: 19054193

[30] Ni M Mooney K Richards L Balachandran A Sun M Harriell K . Comparative impacts of Tai Chi, balance training, and a specially-designed yoga program on balance in older fallers. Arch Phys Med Rehabil. (2014) 95:1620–1628.e30. doi: 10.1016/j.apmr.2014.04.022, PMID: 24835753

[31] Nowalk MP Prendergast JM Bayles CM D'Amico FJ Colvin GC. A randomized trial of exercise programs among older individuals living in two long-term care facilities: the FallsFREE program. J Am Geriatr Soc. (2001) 49:859–65. doi: 10.1046/j.1532-5415.2001.49174.x, PMID: 11527475

[32] Penn IW Sung WH Lin CH Chuang E Chuang TY Lin PH. Effects of individualized Tai-Chi on balance and lower-limb strength in older adults. BMC Geriatr. (2019) 19:235. doi: 10.1186/s12877-019-1250-8, PMID: 31455225 PMC6712673

[33] Pluchino A Lee SY Asfour S Roos BA Signorile JF. Pilot study comparing changes in postural control after training using a video game balance board program and 2 standard activity-based balance intervention programs. Arch Phys Med Rehabil. (2012) 93:1138–46. doi: 10.1016/j.apmr.2012.01.023, PMID: 22414490

[34] Son NK Ryu YU Jeong HW Jang YH Kim HD. Comparison of 2 different exercise approaches: Tai Chi versus Otago, in community-dwelling older women. J Geriatr Phys Ther. (2016) 39:51–7. doi: 10.1519/JPT.0000000000000042, PMID: 25760277

[35] Taylor D Hale L Schluter P Waters DL Binns EE McCracken H . Effectiveness of Tai Chi as a community-based falls prevention intervention: a randomized controlled trial. J Am Geriatr Soc. (2012) 60:841–8. doi: 10.1111/j.1532-5415.2012.03928.x, PMID: 22587850

[36] Taylor-Piliae RE Coull BM. Community-based Yang-style Tai Chi is safe and feasible in chronic stroke: a pilot study. Clin Rehabil. (2012) 26:121–31. doi: 10.1177/0269215511419381, PMID: 21937523

[37] Taylor-Piliae RE Hoke TM Hepworth JT Latt LD Najafi B Coull BM. Effect of Tai Chi on physical function, fall rates and quality of life among older stroke survivors. Arch Phys Med Rehabil. (2014) 95:816–24. doi: 10.1016/j.apmr.2014.01.001, PMID: 24440643

[38] Tousignant M Corriveau H Roy PM Desrosiers J Dubuc N Hebert R. Efficacy of supervised Tai Chi exercises versus conventional physical therapy exercises in fall prevention for frail older adults: a randomized controlled trial. Disabil Rehabil. (2013) 35:1429–35. doi: 10.3109/09638288.2012.737084, PMID: 23167499

[39] Voukelatos A Cumming RG Lord SR Rissel C. A randomized, controlled trial of Tai Chi for the prevention of falls: the Central Sydney Tai Chi trial. J Am Geriatr Soc. (2007) 55:1185–91. doi: 10.1111/j.1532-5415.2007.01244.x, PMID: 17661956

[40] Woo J Hong A Lau E Lynn H. A randomised controlled trial of Tai Chi and resistance exercise on bone health, muscle strength and balance in community-living elderly people. Age Ageing. (2007) 36:262–8. doi: 10.1093/ageing/afm005, PMID: 17356003

[41] Wayne PM Walsh JN Taylor-Piliae RE Wells RE Papp KV Donovan NJ . Effect of Tai Chi on cognitive performance in older adults: systematic review and meta-analysis. J Am Geriatr Soc. (2014) 62:25–39. doi: 10.1111/jgs.12611, PMID: 24383523 PMC4055508

[42] Wayne PM Lee MS Novakowski J Osypiuk K Ligibel J Carlson LE . Tai Chi and Qigong for cancer-related symptoms and quality of life: a systematic review and meta-analysis. J Cancer Surviv. (2018) 12:256–67. doi: 10.1007/s11764-017-0665-5, PMID: 29222705 PMC5958892

[43] Kong LJ Lauche R Klose P Bu JH Yang XC Guo CQ . Tai Chi for chronic pain conditions: a systematic review and Meta-analysis of randomized controlled trials. Sci Rep. (2016) 6:25325. doi: 10.1038/srep25325, PMID: 27125299 PMC4850460

[44] Huang ZG Feng YH Li YH Lv CS. Systematic review and meta-analysis: Tai Chi for preventing falls in older adults. BMJ Open. (2017) 7:e013661. doi: 10.1136/bmjopen-2016-013661, PMID: 28167744 PMC5293999

[45] Kendrick D Kumar A Carpenter H Zijlstra GA Skelton DA Cook JR . Exercise for reducing fear of falling in older people living in the community. Cochrane Database Syst Rev. (2014) 2015:CD009848. doi: 10.1002/14651858.CD009848.pub2, PMID: 25432016 PMC7388865

[46] Zhang JG Ishikawa-Takata K Yamazaki H Morita T Ohta T. The effects of Tai Chi Chuan on physiological function and fear of falling in the less robust elderly: an intervention study for preventing falls. Arch Gerontol Geriatr. (2006) 42:107–16. doi: 10.1016/j.archger.2005.06.007, PMID: 16125805

[47] Hong Y Li JX Robinson PD. Balance control, flexibility, and cardiorespiratory fitness among older Tai Chi practitioners. Br J Sports Med. (2000) 34:29–34. doi: 10.1136/bjsm.34.1.29, PMID: 10690447 PMC1724150

[48] Au-Yeung SS Hui-Chan CW Tang JC. Short-form Tai Chi improves standing balance of people with chronic stroke. Neurorehabil Neural Repair. (2009) 23:515–22. doi: 10.1177/1545968308326425, PMID: 19129308

[49] Lan C Lai JS Chen SY Wong MK. Tai Chi Chuan to improve muscular strength and endurance in elderly individuals: a pilot study. Arch Phys Med Rehabil. (2000) 81:604–7. doi: 10.1016/s0003-9993(00)90042-x, PMID: 10807099

[50] Song QH Zhang QH Xu RM Ma M Zhao XP Shen GQ . Effect of Tai-Chi exercise on lower limb muscle strength, bone mineral density and balance function of elderly women. Int. J Clin Exp Med. (2014) 7:1569–76. PMID: 25035781 PMC4100967

[51] Li B Tang H He G Jin Z He Y Huang P . Tai Chi enhances cognitive training effects on delaying cognitive decline in mild cognitive impairment. Alzheimers Dement. (2023) 19:136–49. doi: 10.1002/alz.12658, PMID: 35290704

[52] Laudisio A Giovannini S Finamore P Loreti C Vannetti F Coraci D . Muscle strength is related to mental and physical quality of life in the oldest old. Arch Gerontol Geriatr. (2020) 89:104109. doi: 10.1016/j.archger.2020.104109, PMID: 32460125

[53] Tsang WW Hui-Chan CW. Effect of 4- and 8-wk intensive Tai Chi training on balance control in the elderly. Med Sci Sports Exerc. (2004) 36:648–57. doi: 10.1249/01.mss.0000121941.57669.bf, PMID: 15064593

[54] Chiesa A Calati R Serretti A. Does mindfulness training improve cognitive abilities? A systematic review of neuropsychological findings. Clin Psychol Rev. (2011) 31:449–64. doi: 10.1016/j.cpr.2010.11.003, PMID: 21183265

[55] Kumar A Delbaere K Zijlstra GA Carpenter H Iliffe S Masud T . Exercise for reducing fear of falling in older people living in the community: Cochrane systematic review and meta-analysis. Age Ageing. (2016) 45:345–52. doi: 10.1093/ageing/afw036, PMID: 27121683

[56] Zheng G Xia R Zhou W Tao J Chen L. Aerobic exercise ameliorates cognitive function in older adults with mild cognitive impairment: a systematic review and meta-analysis of randomised controlled trials. Br J Sports Med. (2016) 50:1443–50. doi: 10.1136/bjsports-2015-095699, PMID: 27095745

[57] Irwin MR Olmstead R Breen EC Witarama T Carrillo C Sadeghi N . Tai Chi, cellular inflammation, and transcriptome dynamics in breast cancer survivors with insomnia: a randomized controlled trial. J Natl Cancer Inst Monogr. (2014) 2014:295–301. doi: 10.1093/jncimonographs/lgu028, PMID: 25749595 PMC4411534

[58] Oh B Butow PN Mullan BA Clarke SJ Beale PJ Pavlakis N . Effect of medical qigong on cognitive function, quality of life, and a biomarker of inflammation in cancer patients: a randomized controlled trial. Support Care Cancer. (2012) 20:1235–42. doi: 10.1007/s00520-011-1209-6, PMID: 21688163

[59] Day L Finch CF Harrison JE Hoareau E Segal L Ullah S. Modelling the population-level impact of Tai-Chi on falls and fall-related injury among community-dwelling older people. Inj Prev. (2010) 16:321–6. doi: 10.1136/ip.2009.025452, PMID: 20643871

[60] Giovannini S Iacovelli C Brau F Loreti C Fusco A Caliandro P . RObotic-assisted rehabilitation for balance and gait in stroke patients (ROAR-S): study protocol for a preliminary randomized controlled trial. Trials. (2022) 23:872. doi: 10.1186/s13063-022-06812-w, PMID: 36224575 PMC9558956

