# Supplementary material for: Tai Chi for fall prevention and balance improvement in older adults: a systematic review and meta-analysis of randomized controlled trials
Source: Front Public Health. 2023 Sep 1;11:1236050. doi: 10.3389/fpubh.2023.1236050 (PMC10509476; doi:10.3389/fpubh.2023.1236050)
Supplement: Supplementary file 1 [file Table_1.DOCX]

**Title: Tai Chi for Fall Prevention and Balance Improvement in Older Adults: A Systematic Review and Meta-Analysis of Randomized Controlled Trials**

**Supplementary table 1. Literature search strategy for all the databases**

Database(s): **Pubmed** From database establishment to 2022.12.31

| **#** | **Searches** | **Results** |
| --- | --- | --- |
| 1 | ("tai ji"[Title/Abstract] OR "tai chi"[Title/Abstract] OR "chi tai"[Title/Abstract] OR "tai ji quan"[Title/Abstract] OR "ji quan tai"[Title/Abstract] OR "quan tai ji"[Title/Abstract] OR "Taiji"[Title/Abstract] OR "Taijiquan"[Title/Abstract] OR "t ai chi"[Title/Abstract] OR "tai chi chuan"[Title/Abstract] OR "tai ji"[MeSH Terms]) | 2547 |
| 2 | ("Elderly"[Title/Abstract] OR "Adult"[Title/Abstract] OR "older adults"[Title/Abstract] OR "old people"[Title/Abstract] OR "senior citizens"[Title/Abstract] OR "Aged"[MeSH Terms]) | 4282785 |
| 3 | ("randomized controlled trial"[Publication Type] OR "randomized"[Title/Abstract] OR "placebo"[Title/Abstract]) | 987773 |
| 4 | 1 AND 2 AND 3 | 524 |

Database(s): **Embase** From database establishment to 2022.12.31

| **#** | **Searches** | **Results** |
| --- | --- | --- |
| 1 | 'tai chi'/exp | 3823 |
| 2 | 'tai chi chuan':ab,ti OR 't ai chi':ab,ti OR 'taijiquan':ab,ti OR 'taiji':ab,ti OR 'quan tai ji':ab,ti OR 'ji quan tai':ab,ti OR 'tai ji quan':ab,ti OR 'chi tai':ab,ti OR 'tai chi':ab,ti OR 'tai ji':ab,ti | 3369 |
| 3 | 1 OR 2 | 4429 |
| 4 | 'aged'/exp | 3649561 |
| 5 | 'elderly':ab,ti OR 'adult':ab,ti OR 'older adults':ab,ti OR 'old people':ab,ti OR 'senior citizens':ab,ti | 1658569 |
| 6 | #4 OR #5 | 4792239 |
| 7 | 'randomized controlled trial':ab,ti OR 'randomized':ab,ti OR 'placebo':ab,ti | 1091143 |
| 8 | #3 AND #6 AND #7 | 534 |

Database(s): **[Cochrane](http://www.baidu.com/link?url=0cLW9bJctMw7gSvc9ZxQjgchACzUVa6MTnb-HO5kjgaDTLt3cES3vWUxPLvwxCsg)** From database establishment to 2022.12.31

| **#** | **Searches** | **Results** |
| --- | --- | --- |
| 1 | MeSH descriptor: [Tai Ji] explode all trees | 492 |
| 2 | ((Quan, Tai Ji):ti,ab,kw OR (Tai Chi Chuan):ti,ab,kw OR (Ji Quan, Tai):ti,ab,kw OR (Tai Chi):ti,ab,kw OR (T'ai Chi):ti,ab,kw OR (Tai-ji):ti,ab,kw OR (Chi, Tai):ti,ab,kw OR (Taiji):ti,ab,kw OR (Taijiquan):ti,ab,kw) (Word variations have been searched) | 1830 |
| 3 | 1 OR 2 | 1830 |
| 4 | MeSH descriptor: [Aged] explode all trees | 255190 |
| 5 | (Elderly):ti,ab,kw OR (Older Adults):ti,ab,kw OR (Older Adult):ti,ab,kw OR (Adult, Older):ti,ab,kw OR (Elder):ti,ab,kw OR (Elders):ti,ab,kw OR (Elderly):ti,ab,kw OR (old people):ti,ab,kw OR (senior citizens):ti,ab,kw OR (senior citizens):ti,ab,kw | 121582 |
| 6 | #4 OR #5 | 340496 |
| 7 | #3 AND #6 | 879 |

**Supplementary table 2. Number of falls between the Tai Chi group and the control group.**

|  | **Tai ji group** | | |  | **Control group** | | | **IRR** |
| --- | --- | --- | --- | --- | --- | --- | --- | --- |
|  | **Number of Falls (n)** | **Number of People** | **Incidence Rate** |  | **Number of Falls** | **Number of People** | **Incidence Rate** |  |
| Total | 1816 | 2539 | 0.72 |  | 2681 | 2475 | 1.08 | 0.66 |
| Day et al. 2015(19) | 150 | 204 | 0.74 |  | 168 | 205 | 0.82 | 0.90 |
| Faber et al. 2006(20) | 96 | 90 | 1.07 |  | 115 | 78 | 1.47 | 0.72 |
| Faber et al. 2006-2(20) | 96 | 90 | 1.07 |  | 108 | 60 | 1.8 | 0.59 |
| Gao et al. 2014(21) | 11 | 37 | 0.30 |  | 25 | 39 | 0.64 | 0.46 |
| Huang et al. 2011(23) | 3 | 56 | 0.05 |  | 8 | 60 | 0.13 | 0.40 |
| Hwang et al. 2016(24) | 55 | 182 | 0.30 |  | 105 | 175 | 0.6 | 0.50 |
| Li et al. 2005(26) | 38 | 95 | 0.4 |  | 73 | 93 | 0.78 | 0.51 |
| Li et al. 2012(8) | 62 | 65 | 0.95 |  | 186 | 65 | 2.86 | 0.33 |
| Li et al. 2012-2(8) | 62 | 65 | 0.95 |  | 133 | 65 | 2.05 | 0.46 |
| Li et al. 2018(27) | 152 | 224 | 0.68 |  | 363 | 223 | 1.63 | 0.42 |
| Li et al. 2018-2(27) | 152 | 224 | 0.68 |  | 218 | 223 | 0.98 | 0.69 |
| Li et al. 2021(28) | 15 | 15 | 1 |  | 26 | 15 | 1.73 | 0.58 |
| Logghe et al. 2009(29) | 115 | 138 | 0.83 |  | 90 | 131 | 0.69 | 1.21 |
| Taylor et al. 2012a(36) | 412 | 233 | 1.77 |  | 350 | 231 | 1.52 | 1.17 |
| Taylor et al. 2012a-2(36) | 132 | 220 | 0.6 |  | 350 | 231 | 1.52 | 0.40 |
| Taylor et al. 2014(37) | 5 | 30 | 0.17 |  | 15 | 28 | 0.54 | 0.31 |
| Taylor et al. 2014-2(37) | 5 | 30 | 0.17 |  | 14 | 31 | 0.45 | 0.37 |
| Tousignant et al. 2013(38) | 42 | 49 | 0.86 |  | 38 | 44 | 0.86 | 0.99 |
| Voukelatos et al. 2007(39) | 86 | 347 | 0.25 |  | 126 | 337 | 0.37 | 0.66 |
| Wolf et al. 2003(7) | 127 | 145 | 0.88 |  | 170 | 141 | 1.21 | 0.73 |

IRR, incidence rate ratio

**Supplementary table 3. Subgroup analysis of falls**

| **Subgroup** | **Included studies** | **Tai chi group** | **Control group** | **Heterogeneity (I^2^)** | **MD [95% CI]** | **p** | **Test for subgroup difference** |
| --- | --- | --- | --- | --- | --- | --- | --- |
| Exposure Time (h) |  |  |  |  |  |  | 0.36 |
| ≤ 24 | 2(24, 39) | 529 | 512 | 84% | -0.17 [-0.40, 0.06] | 0.15 |  |
| 24~48 | 4(21, 23, 27, 38) | 590 | 589 | 69% | -0.33 [-0.60, -0.06] | 0.02 |  |
| >48 | 3(7, 20, 28) | 340 | 294 | 0 | -0.42 [-0.68, -0.15] | 0.002 |  |
| Tai Chi style |  |  |  |  |  |  | <0.001 |
| Sun | 1(39) | 347 | 337 | - | -0.06 [-0.14, 0.01] | 0.09 |  |
| Yang | 3(7, 21, 24) | 364 | 355 | 0 | -0.32 [-0.45, -0.19] | <0.001 |  |
| Weekly frequency |  |  |  |  |  |  | 0.25 |
| Once | 2(24, 39) | 529 | 512 | 84% | -0.17 [-0.40, 0.06] | 0.15 |  |
| Twice | 4(7, 20, 27, 38) | 822 | 769 | 0 | -0.41 [-0.60, -0.23] | <0.001 |  |
| ≥ Three times | 3(21, 23, 28) | 108 | 114 | 60% | -0.24 [-0.53, 0.04] | 0.09 |  |
| Risk of fall |  |  |  |  |  |  | 0.003 |
| Non-high risk | 4(20, 23, 28, 39) | 598 | 550 | 0 | -0.07 [-0.13, -0.01] | 0.02 |  |
| History of falls only | 3(7, 24, 27) | 775 | 762 | 43% | -0.34 [-0.57, -0.20] | <0.001 |  |
| Falls related diseases | 2(21, 38) | 86 | 83 | 0 | -0.34 [-0.65, -0.04] | 0.03 |  |
| Follow-up time |  |  |  |  |  |  | 0.52 |
| ≤ 6 months | 5(20, 23, 27, 28, 39) | 1046 | 996 | 62% | -0.21 [-0.37, -0.05] | 0.01 |  |
| 6~12 months | 3(7, 21, 38) | 231 | 224 | 0 | -0.36 [-0.57, -0.15] | <0.001 |  |
| >12 months | 1(24) | 182 | 175 | - | -0.30 [-0.47, -0.13] | <0.001 |  |

MD, mean difference; CI, confidence interval.

**Supplementary table 4. Subgroup analysis of TUG**

| **Subgroup** | **Included studies** | **Tai chi group total** | **Control group total** | **Heterogeneity (I^2^)** | **MD [95% CI]** | **p** | **Test for subgroup difference** |
| --- | --- | --- | --- | --- | --- | --- | --- |
| Exposure Time (h) |  |  |  |  |  |  | 0.02 |
| ≤ 24 | 6(25, 30, 32-34, 36) | 317 | 322 | 83% | 0.03 [-0.67, 0.73] | 0.93 |  |
| 24~48 | 4(8, 21, 27, 36) | 835 | 846 | 68% | -0.92 [-1.63, -0.21] | 0.01 |  |
| >48 | 3(22, 26, 28) | 186 | 208 | 86% | -1.29 [-1.93, -0.65]] | <0.001 |  |
| Tai Chi style |  |  |  |  |  |  | 0.01 |
| Sun | 4(25, 33, 34, 36) | 511 | 523 | 79% | 0.16 [-0.53, 0.84] | 0.66 |  |
| Yang | 3(21, 26, 32) | 171 | 193 | 0 | -0.74 [-0.92, -0.55] | <0.001 |  |
| Weekly frequency |  |  |  |  |  |  | 0.006 |
| Once | 1(36) | 233 | 231 | - | -0.10 [-0.74, 0.54]] | 0.76 |  |
| Twice | 7(8, 25, 27, 30, 33, 34, 36) | 867 | 883 | 81% | -0.31 [-0.88, 0.25] | 0.28 |  |
| ≥ Three times | 5(21, 22, 26, 28, 32) | 238 | 262 | 80% | -1.41 [-2.01, -0.80]] | <0.001 |  |
| Risk of fall |  |  |  |  |  |  | 0.45 |
| Non-high risk | 7(22, 25, 26, 28, 32-34) | 259 | 284 | 91% | -0.68 [-1.38, 0.02] | 0.06 |  |
| History of falls only | 3(27, 30, 36) | 912 | 923 | 61% | -0.46 [-0.95, 0.03] | 0.07 |  |
| Falls related diseases | 2(8, 21) | 167 | 169 | 58% | -1.13 [-2.07, -0.19] | 0.02 |  |
| Follow-up time |  |  |  |  |  |  | 0.13 |
| ≤ 3 months | 5(25, 30, 32-34) | 84 | 91 | 86% | 0.04 [-0.86, 0.94] | 0.93 |  |
| 3~6 months | 5(8, 26-28, 36) | 1186 | 1199 | 78% | -0.95 [-1.50, -0.41] | <0.001 |  |
| 6~12 months | 2(21, 22) | 68 | 86 | 79% | -1.26 [-2.59, 0.08] | 0.06 |  |

TUG, timed up and go; MD, mean difference; CI, confidence interval.

**Supplementary table 5. Subgroup analysis of FRT**

| **Subgroup** | **Included studies** | **Tai chi group total** | **Control group total** | **Heterogeneity (I^2^)** | **MD [95% CI]** | **p** | **Test for subgroup difference** |
| --- | --- | --- | --- | --- | --- | --- | --- |
| Exposure Time (h) |  |  |  |  |  |  | 0.56 |
| ≤ 24 | 5(25, 30, 32-34) | 97 | 105 | 33% | 2.41 [1.19, 3.64] | <0.001 |  |
| 24~48 | 2(8, 27) | 578 | 576 | 91% | 1.92 [0.57, 3.27] | 0.005 |  |
| >48 | 2(22, 26) | 156 | 178 | 99% | 4.86 [-0.67, 10.38] | 0.09 |  |
| Tai Chi style |  |  |  |  |  |  | 0.13 |
| Sun | 3(25, 33, 34) | 58 | 61 | 0 | 0.26 [-2.02, 2.55] | 0.82 |  |
| Yang | 2(26, 32) | 140 | 146 | 0 | 2.08 [1.45, 2.70] | <0.001 |  |
| Weekly frequency |  |  |  |  |  |  | 0.33 |
| Once | - | - | - | - | - | - | - |
| Twice | 6(8, 25, 27, 30, 33, 34) | 638 | 667 | 85% | 2.06 [1.00, 3.11] | <0.001 |  |
| ≥ Three times | 2(22, 26) | 156 | 178 | 99% | 4.86 [-0.67, 10.38] | 0.09 |  |
| Risk of fall |  |  |  |  |  |  | 0.30 |
| Non-high risk | 6(22, 25, 26, 32-34) | 229 | 254 | 96% | 2.74 [-0.58, 6.06] | 0.11 |  |
| History of falls only | 2(27, 30) | 470 | 476 | 93% | 1.95 [0.65, 3.25] | 0.003 |  |
| Falls related diseases | 1(8) | 130 | 130 | 6% | 2.69 [1.98, 5.11] | <0.001 |  |
| Follow-up time |  |  |  |  |  |  | <0.001 |
| ≤ 3 months | 5(25, 30, 32-34) | 95 | 106 | 17% | 2.64 [1.60, 3.67] | <0.001 |  |
| 3~6 months | 3(8, 26, 27) | 703 | 707 | 91% | 1.90 [0.83, 2.98] | <0.001 |  |
| 6~12 months | 1(22) | 31 | 47 | - | 7.68 [6.93, 8.43] | <0.001 |  |

FRT, functional reach test; MD, mean difference; CI, confidence interval.
