# Supplementary figures and images for: Tai Chi for fall prevention and balance improvement in older adults: a systematic review and meta-analysis of randomized controlled trials
Source: Front Public Health. 2023 Sep 1;11:1236050. doi: 10.3389/fpubh.2023.1236050 (PMC10509476; doi:10.3389/fpubh.2023.1236050)

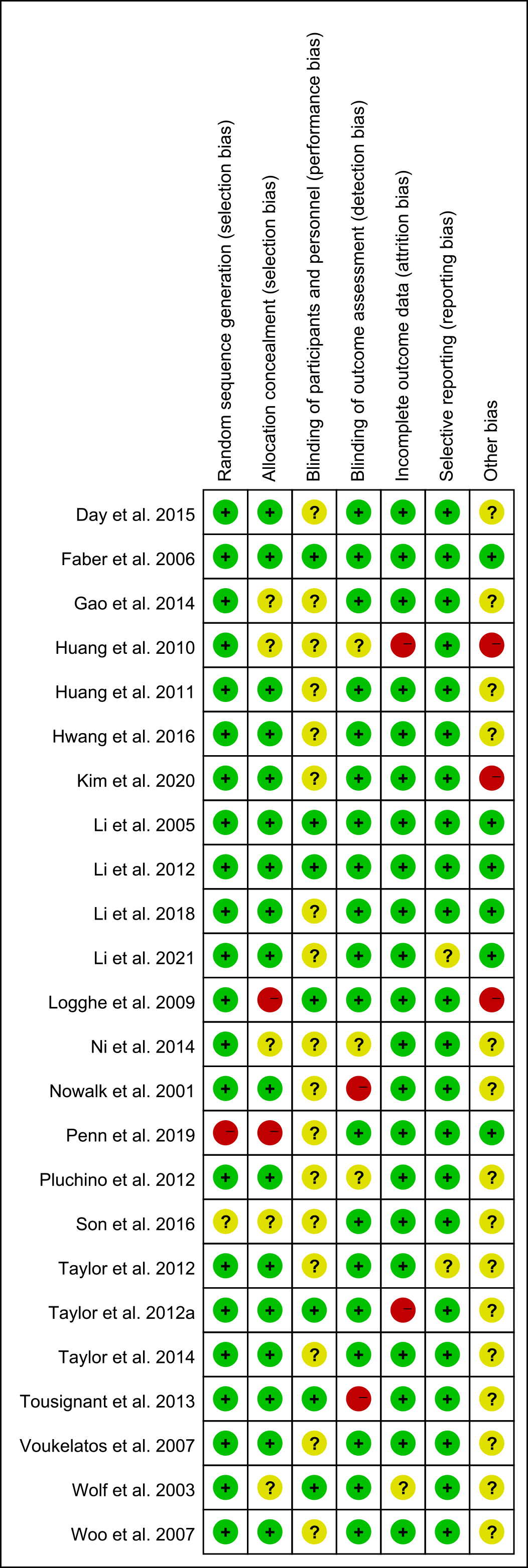

Supplement: Supplementary Figure 1 — Risk of bias summary. [file Image_1.TIF]
